# Young and Undamaged rMSA Improves the Healthspan and Lifespan of Mice

**DOI:** 10.3390/biom11081191

**Published:** 2021-08-12

**Authors:** Jiaze Tang, Anji Ju, Boya Li, Shaosen Zhang, Yuanchao Gong, Boyuan Ma, Yi Jiang, Hongyi Liu, Yan Fu, Yongzhang Luo

**Affiliations:** 1 The National Engineering Laboratory for Anti-Tumor Protein Therapeutics, Tsinghua University, Beijing 100084, China; tjz15@mails.tsinghua.edu.cn (J.T.); jaj16@mails.tsinghua.edu.cn (A.J.); liby15@mails.tsinghua.edu.cn (B.L.); zhangss14@tsinghua.org.cn (S.Z.); gongyuanchao1992@163.com (Y.G.); mby17@mails.tsinghua.edu.cn (B.M.); jiang-y17@mails.tsinghua.edu.cn (Y.J.); liu-hy18@mails.tsinghua.edu.cn (H.L.); 2 Beijing Key Laboratory for Protein Therapeutics, Tsinghua University, Beijing 100084, China; 3 Cancer Biology Laboratory, School of Life Sciences, Tsinghua University, Beijing 100084, China

**Keywords:** rMSA, protein damage, strength, memory, healthspan, lifespan

## Abstract

Improvement of longevity is an eternal dream of human beings. The accumulation of protein damages is considered as a major cause of aging. Here, we report that the injection of exogenous recombinant mouse serum albumin (rMSA) reduced the total damages of serum albumin in C57BL/6N mice, with higher level of free-thiols, lower levels of carbonyls and advanced glycation end-products as well as homocysteines in rMSA-treated mice. The healthspan and lifespan of C57BL/6N mice were significantly improved by rMSA. The grip strength of rMSA-treated female and male mice increased by 29.6% and 17.4%, respectively. Meanwhile, the percentage of successful escape increased 23.0% in rMSA-treated male mice using the Barnes Maze test. Moreover, the median lifespan extensions were 17.6% for female and 20.3% for male, respectively. The rMSA used in this study is young and almost undamaged. We define the concept “young and undamaged” to any protein without any unnecessary modifications by four parameters: intact free thiol (if any), no carbonylation, no advanced glycation end-product, and no homocysteinylation. Here, “young and undamaged” exogenous rMSA used in the present study is much younger and less damaged than the endogenous serum albumin purified from young mice at 1.5 months of age. We predict that undamaged proteins altogether can further improve the healthspan and lifespan of mice.

## 1. Introduction

Longevity is an eternal pursuit of human beings. Tales of passionate seeking for immortality run through the whole human history. Ludwig et al. reported the extended lifespan of older rats by younger rats in the parabiosis model for the first time in 1972 [1]. Egerman group and Villeda group, respectively, found that the muscle strength and cognitive ability of old mice were improved after the parabiosis surgery with young mice [2,3], which suggest that the “mystery” of aging may exist in blood proteins. It is believed that aging is at least partially caused by the continuous accumulation of damages or unnecessary modifications of proteins [4,5,6], including free thiol oxidation, carbonylation, advanced glycation end-product (AGE) formation, and homocysteinylation [7,8,9,10].

Human serum albumin (HSA, UniProtKB P02768) is the most abundant protein in blood plasma with a serum half-life of about 21 days [11]. Damages or unnecessary modifications of HSA are related to many pathological conditions and increase with age. Firstly, the single free thiol in Cys-34 residue of HSA has been proposed to account for approximately 80% of the total free thiols in plasma [12,13], whose oxidation is intimately linked with aging and age-related diseases [14,15,16]. Secondly, in oxidative environments, carbonyls are also formed especially on the side chains of Pro, Arg, Lys and Thr residues in proteins [17,18]. Elevated carbonyl levels in HSA have been found to be related to aging and varieties of diseases [19,20,21]. Thirdly, the AGE accumulation of HSA is another important factor found to be involved in aging [9,22]. It is widely reported that AGE formation impairs normal functions of albumin and can induce inflammatory responses, which is connected with aging and the progression of serious diseases [22,23]. Fourthly, it has been widely reported that homocysteine (Hcy) increases with age and is associated with age-related degenerative disorders [10,24,25,26]. HSA is a major target for homocysteinylation, thus it can efficiently protect other proteins from the toxicity of Hcy [27,28,29].

Therefore, treatment of freshly prepared recombinant serum albumin with no damages or unnecessary modifications is most likely to extend lifespan and healthspan. Here, we report that young and undamaged recombinant mouse serum albumin (rMSA) -treated groups in C57BL/6N mice obtained significantly extended lifespan with increased skeletal muscle strength and cognitive ability compared with saline-treated groups.

## 2. Materials and Methods

### 2.1. Mice and Drug Treatments

C57BL/6N mice were purchased from Beijing Vital River Laboratory Animal Technology Co., Ltd. (a distributor of Charles River Laboratories, Beijing, China). The mice transport stress syndrome was carefully avoided during the transportation to the Laboratory Animal Research Center, Tsinghua University (THU-LARC). All mice were quarantined for one month to guarantee the adaptation to the new environment and carried out quality inspection. Animals were kept in a pathogen-free barrier environment with a 12-h dark-light circle. Room temperature was maintained at 23 °C. After arrival, mice were fed with irradiation-sterilized JAX-standard breeder chow (SHOOBREE^®^, Xietong Pharmaceutical Bio-technology Co., Ltd., Jiangsu, China, 1010058) and sterilized water during the entire study.

12-month-old middle aged mice were divided into rMSA- or saline-treated group randomly. More than one kilogram correctly refolded rMSA was kindly supplied by Shenzhen Protgen, Ltd. (Shenzhen, China). The quality of GMP-grade rMSA, expressed by *pichia pastoris*, was strictly controlled to ensure that the purity is greater than 99%. Most importantly, host cell proteins (HCPs) were less than 1 μg/g rMSA by ELISA, which means our rMSA is almost free of HCPs.

Then, 125 mg/mL of rMSA dissolved in saline was i.v. injected slowly. Mice were weighed before each injection to calculate the dosage, with saline serving as the negative control. Mice were injected with 1.5 mg rMSA per gram of mouse body weight and isometric saline every 3 weeks as indicated. All animal studies were approved by the Institutional Animal Care and Use Committee of Tsinghua University (Beijing, China).

### 2.2. Protein Levels Determination

To determine the blood biochemical parameters, blood samples were collected from mouse orbital sinus after Avertin^®^ (Tribromoethanol, Sigma-Aldrich, St. Louis, MO, USA, T48402) intraperitoneal injection (400 mg/kg) for anesthesia. Serum samples were collected after centrifugation at 1000× *g* for 20 min at 4 °C. To collect plasma samples, heparin sodium salt is added to the fresh blood samples (20 units/mL blood, Sigma-Aldrich, H3149) to prevent blood clotting followed by centrifugation at 1000× *g* for 30 min at 4 °C. Major blood biochemical parameters of serum samples were determined with an automatic biochemistry analyzer (Olympus AU 400).

To determine the expression level of albumin, mice were euthanized using carbon dioxide after anesthesia. Liver tissue samples were quickly removed and homogenized. The total RNA from the homogenate was isolated using TRIzol Reagent (Invitrogen, Pittsburgh, PA, USA, 15596026) and converted into cDNA using the First Strand cDNA Synthesis Kit (Fermentas, Hanover, NH, USA, K1622). Quantitative RT-PCR (qRT-PCR) was performed using the TransStart^®^ Top Green qPCR SuperMix (TransGen Biotech Co., Beijing, China, AQ131). Relative quantitation was analyzed using the ΔΔ*Ct* method. Glyceraldehyde 3-phosphate dehydrogenase (GAPDH) was used as an internal control. Independent experiments were repeated in triplicates. The following primers were used: *Alb* forward 5′-TGCTTTTCCAGGGGTGTGTT, reverse 5′-TTACTTCCTGCACTAATTTGGCA; *Gapdh* forward 5′-GTTGTCTCCTGCGACTTCA, reverse 5′-GGTGGTCCAGG GTTTCTTA.

### 2.3. Grip Strength Test

The grip strength was measured using a grip strength meter (Yiyan Co. Ltd., Shanghai, China, YLS-13A). Mice were allowed to hold onto a metal grid and were gently pulled backwards by the tail at a constant speed until the mice could no longer hold the grid. Each mouse was given five trials, and the average value was used to represent the grip strength of an individual mouse. The experiments were carried out in a randomized double-blind procedure.

### 2.4. Barnes Maze Assay

Male mice treated with rMSA or isometric saline for 8 months were subjected to the Barnes maze assay to evaluate spatial memory function. For the Barnes maze assay, mice were trained to find a hole that connected to a black escape box, which was positioned around the circumference of a circular platform (Shanghai XinRuan, Shanghai, China, XR-XB108). The circular platform was 91 cm diameter and 0.4 cm thick, with 20 evenly distributed 5 cm diameter holes around the edge, with two overhead lights served as an aversive stimulus. Each trial was recorded by a video camera installed over the platform. Procedures were similar as described by Rosenfeld et al. with modifications [30]. The results were analyzed by Super Maze software. The experiments were carried out in a randomized double-blind procedure.

### 2.5. Albumin Purification

Serum samples of indicated groups were diluted with 20 mM Tris buffer containing 0.15 M NaCl at pH 7.8 before applying to a pre-equilibrated Blue BestaroseTMFF column (Bestchrom, Shanghai, China), followed by 3-bed volumes wash of nonspecific binding proteins. Mouse albumin was eluted by elution buffer (0.2 M NaSCN, pH 8.0), then dialyzed against PBS and concentrated by Amicon^®^ ultra centrifugal filters with Ultracel-100 and Ultracel-50 regenerated cellulose membrane (MerckMillipore, Darmstadt, Germany, UFC810008, UFC805008) at 4 °C. Protein concentrations were determined by the Pierce™ BCA Protein Assay Kit according to manufacturer’s instructions (Thermo Scientific, 23227). Samples were analyzed on a Quadrupole-Time of Flight (Q-TOF) mass spectrometer (Waters, Milford, MA, USA, SYNAPT G2-Si) instrument optimized for high-mass protein molecular weight analysis.

### 2.6. Immunofluorescence Assay

Frozen sections of mice were dissected from mice and fixed with cold acetone. Then, these samples were blocked with 10% goat serum and stained with primary antibodies overnight at 4 °C followed by the appropriate secondary fluorescently labeled antibodies at 4 °C overnight. Slides were stained with FITC-conjugated secondary antibodies, and nuclei were stained by 4′,6-diamidino-2-phenylindole (DAPI). Fluorescence imaging was performed on a Nikon A1 laser scanning confocal microscope and was analyzed with NIS-Elements Software (Nikon, Tokyo, Japan) and ImageJ software.

The following antibodies were used: mouse monoclonal antibody against phosphorylated microtubule-associated protein tau (p-tau, UniProtKB P10637. Waltham, WA, USA, MN1020), mouse monoclonal antibody against slow myosin heavy chain I (MYH1, UniProtKB Q5SX40. Sigma-Aldrich, St. Louis, MO, USA, M8421), rabbit monoclonal antibody against α-smooth muscle actin (α-SMA, UniProtKB P62737. Cell Signaling Technology, Danvers, MA, USA, 19245), FITC-conjugated goat polyclonal antibody against mouse IgG (H+L) (Abcam, ab6785), and FITC-conjugated goat polyclonal antibody against rabbit IgG (H+L) (Abcam, Cambridge, UK, ab97050).

### 2.7. Masson’s Trichrome Staining

Paraformaldehyde-fixed, paraffin-embedded tissue sections from mice were deparaffinized and rehydrated. Then, sections were stained with the Masson’s Trichrome Stain Kit (KeyGEN BioTECH, Nanjing, China, KGMST-8004). Nuclei stain black, cytoplasm and muscle fibers stain red, and collagen displays a blue coloration.

### 2.8. Toluidine Blue O Staining

Paraformaldehyde-fixed, paraffin-embedded tissue sections from mice were deparaffinized and rehydrated. Then, sections were stained with the Toluidine Blue O reagent according to the manufacturer’s instructions (Solarbio, Beijing, China, G3668).

### 2.9. Immunohistochemical Assay

The rehydrated sections were rinsed three times with PBS, and the endogenous peroxidase was blocked with 3% H_2_O_2_. Then, the samples were blocked with 10% goat serum and incubated with primary antibodies overnight at 4 °C followed by the appropriate secondary HRP-conjugated antibodies at 4 °C overnight. Slides were stained with a newly prepared DAB substrate, and nuclei were stained by hematoxylin. The immunohistochemical staining intensity was quantified with ImageJ software.

The following antibodies were used: rabbit monoclonal antibody against collagen I (COL1A1, UniProtKB P11087. Cell Signaling Technology, Danvers, MA, USA, 91144), rabbit polyclonal antibody against desmin (UniProtKB P31001. Thermo Fisher Scientific, Waltham, MA, USA, PA5-16705), rabbit monoclonal antibody against α-SMA (Cell Signaling Technology, Danvers, MA, USA, 19245), and HRP-conjugated goat polyclonal antibody against rabbit IgG (H+L) (Abcam, Cambridge, UK, ab205718).

### 2.10. Determination of Protein Damages

The Ellman’s method was used to determine the content of free thiols [31]. Mouse serum albumin (MSA, UniProtKB P07724) and rMSA were mixed with equal volumes of 5, 5′-Dithiobis-(2-nitrobenzoic acid) (DTNB) reagent, respectively. The volume and concentration of DTNB used in this study were 100 μL and 2 mM, respectively. Then, 800 μL Tris buffer (1 M) was added to make the volume of the reaction system reach 1000 μL. Samples were kept at room temperature for 30 min. The fluorescence absorbance was measured at 412 nm. Carbonyls in protein samples were quantified using the Protein Carbonyl Content Assay Kit (Abcam, Cambridge, UK, ab126287) according to the manual. Hcy concentrations were measured by the enzyme-linked immunosorbent assay (ELISA) according to the manufacturer’s instructions (MEIMIAN, Yancheng, China, 1213). Concentrations of AGE were measured with an ELISA kit according to manufacturer’s instructions (CLOUD-CLONE Co., Wuhan, China, CEB353Ge).

### 2.11. Statistical Analysis

The Kaplan–Meier method was used for survival analysis, and the overall survival curves were compared by using the log-rank (Mantel-Cox) test. The variance across samples was analyzed using Kolmogorov–Smirnov (K-S) test and Levene’s test, followed by a 2-tailed unpaired Student *t*-test, where *p* < 0.05 is considered significant. Statistical analysis and diagramming were carried out by the Graphpad Prism 6.01 software unless otherwise noted.

## 3. Results

### 3.1. Exogenous rMSA Treatment Reduced the Damages of Endogenous Albumin

**Figure 1:**
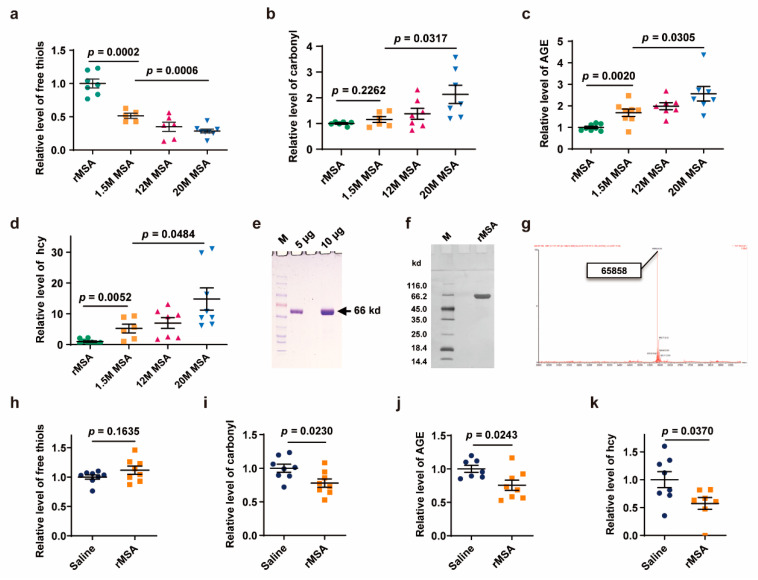
rMSA treatment improved four parameters related to aging. (**a**–**d**) The level of free thiol (**a**), carbonyl (**b**), AGE (**c**), and hcy (**d**) of rMSA and endogenous albumin from serum samples of mice at 1.5-, 12-, and 20 months of age. (**e**–**g**) The molecular weight and protein purity of rMSA were verified by SDS-PAGE with Coomassie Brilliant Blue staining (**e**), silver staining (**f**) and mass spectrometry (**g**). (**h**–**k**) The level of free thiols (**h**), carbonyl (**i**), AGE (**j**), and hcy (**k**) of endogenous albumin of mice treated with body weight-adjusted dosage of rMSA or isometric saline. All graphs represent mean with SEM, with *p* values calculated by the two-tail *t* test.

We proposed for the first time that the status of free thiol, carbonyl, AGE, and hcy can define a young and undamaged protein. To verify this hypothesis, endogenous serum albumin samples of mice at 1.5-, 12-, and 20 months of age were purified, respectively, for comparison. During the aging process, serum albumin undergoes a series of changes in the four parameters: decreased level of free thiol and increased levels of carbonyl, AGE, and hcy. The exogenous rMSA used in this study is even younger and less damaged than endogenous serum albumin from the young mice even at 1.5 months of age. When compared with the endogenous serum albumin from 1.5-month-old mice, the exogenous rMSA contains more free thiols (94.5 % increased, *p* = 0.0002) (Figure 1a), less carbonyl (13.0% decreased, *p* = 0.2262) (Figure 1b), less AGE (40.6% decreased, *p* = 0.0020) (Figure 1c), and less hcy (80.9% decreased, *p* = 0.0052) (Figure 1d). In addition, we need to emphasize here that no other damage was observed in our samples (Figure 1e,f), because the molecular weight measured by mass spectrometry (Figure 1g) is exactly the same as the theoretically calculated value [32]. In sum, exogenous rMSA used in this study is not only “young”, but also almost “undamaged”, which endows rMSA to offer more protection against damages, and suggests that the four parameters could monitor the aging process. Here, “young” means that the exogenous rMSA is much fresher than the endogenous albumin from young mice at the age of only 1.5 months analyzed by the 4 parameters (free thiol, carbonyl, AGE, and hcy). “Undamaged” theoretically means intact free thiol, no AGE, no carbonylation, and no homocysteinylation. In reality, due to the preparation process and detection methods, it is almost impossible to get such perfect sample.

In order to explore whether young and undamaged exogenous rMSA could reduce the damages of albumin in mice, 12-month-old mice were treated with 1.5 mg rMSA per gram of body weight or isometric saline every 3 weeks for 8 months. The albumin was purified from serum samples collected on the 21st day after the last injection. Compared with the saline-treated mice, the albumin from the rMSA-treated mice contained more free thiols (11.6% increased, *p* = 0.1635), much lower levels of carbonyl (22.1% decreased, *p* = 0.0230), AGE (24.4% decreased, *p* = 0.0243), and hcy (42.6% decreased, *p* = 0.0370) (Figure 1h–k). Taken together, young and undamaged exogenous rMSA provides a powerful protective function against oxidation of free thiol, carbonylation, AGE formation, and homocysteinylation.

### 3.2. Exogenous rMSA Enhanced the Function of Skeletal Muscle in Mice

**Figure 2:**
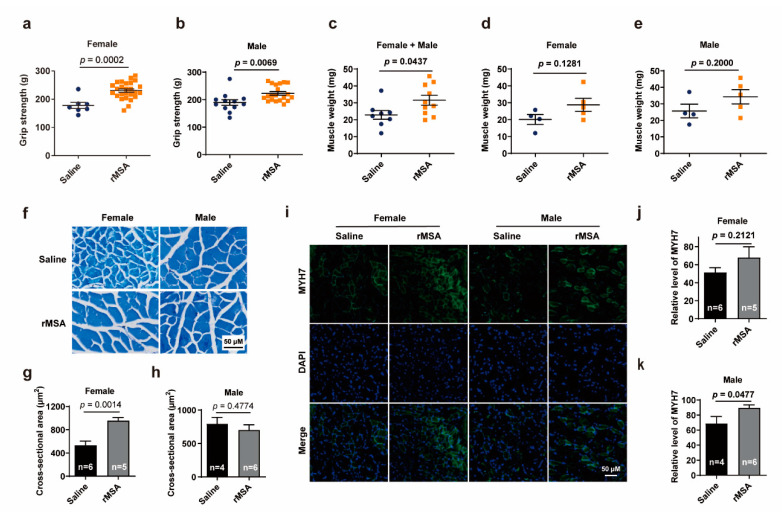
The effects of rMSA on the function of skeletal muscles in mice (**a**,**b**) The grip strength of female (**a**) and male (**b**) mice. (**c**–**e**) The muscle weight of female + male (**c**), female (**d**), and male (**e**) mice. (**f**) The Toluidine Blue O staining of gastrocnemius muscle. Scale bar, 50 μm. (**g**,**h**) The cross-sectional area of myofibers in female (**g**) and male (**h**) mice. (**i**) The immunofluorescence staining for MYH7 (green) and DAPI (blue) in mice. Scale bar, 50 μm. (**j**,**k**) The relative level of MYH7 in female (**j**) and male (**k**) mice. Mice were treated with rMSA 1.5 mg per gram of body weight or isometric saline every 3 weeks for 8 months. All graphs represent mean with SEM, with *p* values calculated by the two-tail *t* test. n, number of mice used for each analysis.

The reduced aging-related albumin damages triggered us to further explore whether the healthspan could be improved. As the dysfunction in skeletal muscle was commonly observed during aging, we first detected the changes of grip strength in mice treated with exogenous rMSA or isometric saline for 8 months. rMSA-treated mice exhibited significantly increased forelimb grip strength from 177.9 g to 230.5 g (29.6% increased, *p* = 0.0002) in females and from 189.6 g to 222.5 g (17.4% increased, *p* = 0.0069) in males, as compared to saline-treated mice (Figure 2a,b).

As the strength of skeletal muscles was largely determined by the muscle mass, we first evaluated the effects of rMSA injection on the in vivo skeletal muscle weights. The gastrocnemius muscle of rMSA-treated mice was heavier than that of the saline-treated mice (Figure 2c), though not significant in the female or male group (Figure 2d,e). We further performed histological analysis on gastrocnemius muscles (Figure 2f). The cross-sectional area of myofibers in rMSA-treated female mice were significantly increased (79.1% increased, *p* = 0.0014) than those in the saline group (Figure 2g). However, a similar phenomenon was not observed in male mice (Figure 2h). Another important parameter to evaluate the muscle strength is the muscular endurance, which is mainly attributed to the type I muscle fibers. We next investigated the expression level of MYH7 (Figure 2i), which is a marker of type I muscle fibers. Male mice treated with rMSA presented significantly more MYH7 positive fibers than saline-treated mice (30.5% increased, *p* = 0.0477, Figure 2k), while similar results were not obtained in female mice (Figure 2j). Taken together, it was demonstrated that exogenous rMSA treatment enhanced the cross-sectional area of gastrocnemius fibers in female mice, and increased the level of MYH7 in male mice. We observed that rMSA had different effects on skeletal muscles of male and female mice, and the variance of hormones and metabolic mechanisms may be one explanation for these differences.

### 3.3. Exogenous rMSA Improved the Spatial Learning Ability and Memory of Mice

**Figure 3:**
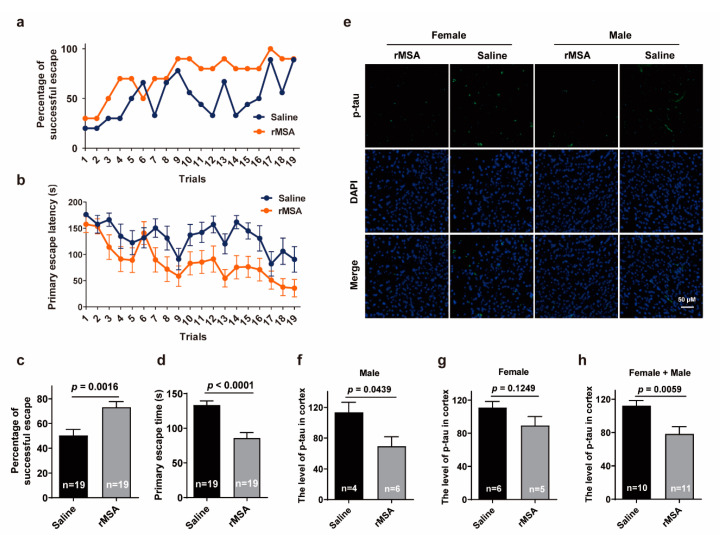
Effects of rMSA on the spatial learning and memory of mice. (**a**,**b**) Measurements of the percentage of successful escape (**a**) and the primary escape latency (**b**) of male mice. (**c**) The average of (**a**). n, the number of trials. (**d**) The average of (**b**). n, the number of trials. (**e**) The representative images of p-tau in the mice cortex. Scale bar, 50 μm. (**f**–**h**) The quantitative results of p-tau (green) and DAPI (blue) in male (**f**), female (**g**) and female + male (**h**) mice. n, the number of mice used for analysis. Mice were treated with rMSA 1.5 mg per gram of body weight or isometric saline every 3 weeks for 8 months. All graphs represent mean with SEM, with *p* values calculated by the two-tail *t* test.

We next investigated the effects of exogenous rMSA on aging-related impairment of memory using the Barnes Maze tests in male mice. rMSA-treated group exhibited a dramatic increase in the percentage of successful escape (73.2% vs. 50.2%, 23.0% increased, *p* = 0.0016) compared to that of the saline-treated group (Figure 3a,c). Meanwhile, the rMSA-treated male mice displayed significantly reduced primary escape latency (85.8 s vs. 133.4 s, 47.6 s faster, *p* < 0.0001) than the saline-treated mice (Figure 3b,d). All these results demonstrated that rMSA treatment significantly improved the ability of spatial learning and memory in aging mice.

We then evaluated the histological changes associated with the memory using these groups of mice. Excitingly, the results of immunofluorescence staining in the cortex showed that the level of phosphorylated-tau (p-tau) was significantly decreased by rMSA treatment in male mice than that of the saline group (39.1% decreased, *p* = 0.0439, Figure 3e,f). However, there was no significant discrepancy in female groups, though the level of p-tau in rMSA-treated mice was lower than that of the saline-treated mice (19.5% decreased, *p* = 0.1249, Figure 3g). In sum, injection of exogenous rMSA decreased the p-tau level of mice (30.1% decreased, *p* = 0.0059, Figure 3h), especially male mice. We propose that the symptoms of age-related neurodegenerative disorders such as the loss of memory and spatial learning abilities can be relieved by rMSA injection.

### 3.4. Exogenous rMSA Treatment Increased the Lifespan in Mice

**Figure 4:**
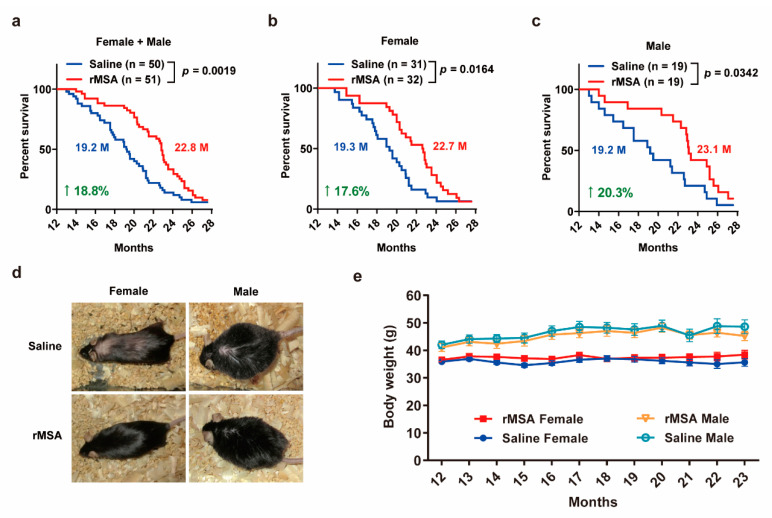
rMSA treatment increased the lifespan in mice. (**a**–**c**) Overall survival curves of female + male (**a**), female (**b**) and male (**c**) mice treated with 1.5 mg rMSA per gram of body weight or isometric saline every 3 weeks. Median survivals (in months, M) and percentage increases are indicated. *p*-value was calculated by the log-rank (Mantel-Cox) test. n, number of mice used for each analysis. (**d**) Representative images of aged mice injected with rMSA or saline. (**e**) Body weight of female and male mice treated with 1.5 mg rMSA per gram of body weight or isometric saline every 3 weeks. All graphs represent mean with SEM.

In order to verify whether exogenous rMSA treatment can extend the lifespan of mice, 12-month-old middle aged C57BL/6N mice were i.v. injected with 1.5 mg rMSA per gram of body weight or isometric saline every 3 weeks until death. The lifespans of rMSA-treated mice were improved significantly (Figure 4a), wherein 17.6% for females (3.4 months increased, *p* = 0.0164, Figure 4b) and 20.3% for males (3.9 months increased, *p* = 0.0342, Figure 4c). Changes in the appearance of both sexes were observed when the median lifespan was reached. Interestingly, mice treated with rMSA had glossier and thicker fur than saline-treated mice (Figure 4d). Moreover, rMSA had no effect on the body weight in both female and male groups (Figure 4e). We noticed that the lifespan of mice varies in different laboratories because of the different feeding conditions. The lifespans of the saline-treated mice in our study were similar to those of the unmanipulated wild type C57BL/6 mice in other studies [33,34]. In sum, exogenous rMSA can extend the medium lifespan of mice.

### 3.5. Exogenous rMSA Treatment Is Safe for C57BL/6N Mice

**Figure 5:**
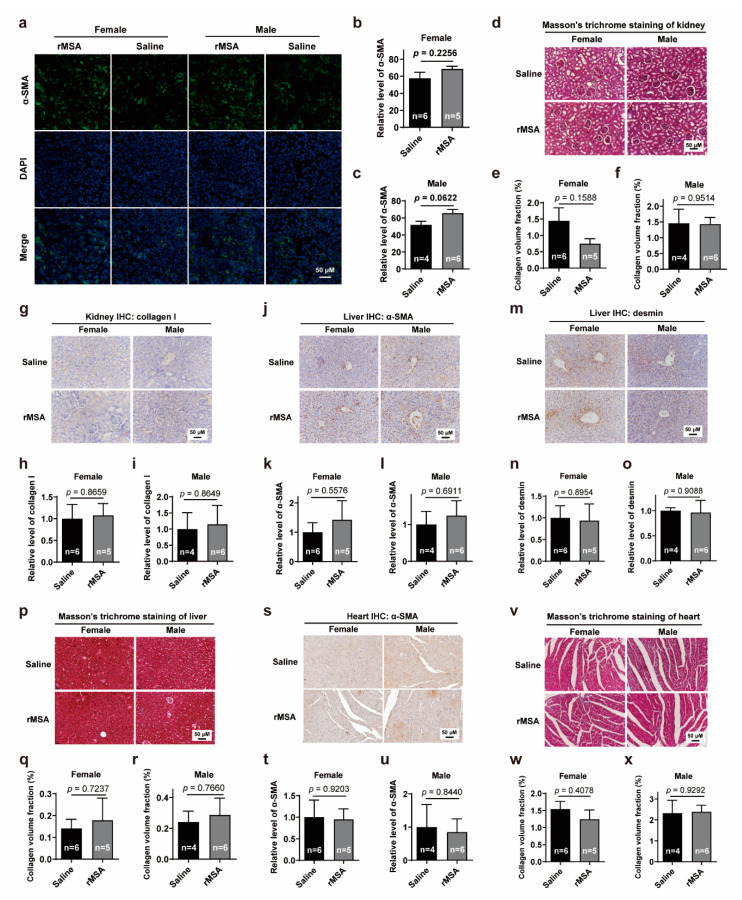
Effects of rMSA injection on the fibrosis level of kidney, liver and heart (**a**) The representative images of α-SMA in mice kidney. Scale bar, 50 μm. (**b**,**c**) The quantitative results of α-SMA level in female (**b**) and male (**c**) mice. (**d**) The Masson’s trichrome staining of mice kidney. Scale bar, 50 μm. (**e**,**f**) The collagen volume fraction of the kidney in female (**e**) and male (**f**) mice. (**g**) The immunohistochemical staining for collagen I in mice kidney. Scale bar, 50 μm. (**h**,**i**) The relative level of collagen I in female (**h**) and male (**i**) mice. (**j**) The immunohistochemical staining for α-SMA in mice liver. Scale bar, 50 μm. (**k**,**l**) The relative level of α-SMA in female (**k**) and male (**l**) mice. (**m**) The immunohistochemical staining for desmin in mice liver. Scale bar, 50 μm. (**n**,**o**) The relative level of desmin in female (**n**) and male (**o**) mice. (**p**) The Masson’s trichrome staining of mice liver. Scale bar, 50 μm. (**q**,**r**) The collagen volume fraction of the liver in female (**q**) and male (**r**) mice. (**s**) The immunohistochemical staining for α-SMA in mice heart. Scale bar, 50 μm. (**t**,**u**) The relative level of α-SMA in female (**t**) and male (**u**) mice. (**v**) The Masson’s trichrome staining of mice cardiac muscle. Scale bar, 50 μm. (**w**,**x**) The collagen volume fraction of the cardiac muscle in female (**w**) and male (**x**) mice. All graphs represent mean with SEM, with *p* values calculated by the two-tail *t* test. n, number of mice used for each analysis.

Moreover, we assessed the safety of exogenous rMSA treatment. qRT-PCR and blood biochemical analyses showed that both mRNA levels in the liver (Figure S1a) and protein (Figure S1b) levels in plasma of albumin underwent slight fluctuations before returning to normal within 8 days after the first injection. Major blood biochemical parameters remained constant in normal levels (Figure S1c–p). In addition, to confirm whether the long-term treatment of saline or rMSA has various degrees of damages to organs, tissue sections of liver, kidney and heart were examined for any histopathological changes (Figure S2a–c). Levels of α-SMA, a marker of myofibroblast activation in organ fibrosis [35], were measured in kidney (Figure 5a–c), which showed no significant difference between saline- and rMSA-treated groups. To further verify the degree of renal fibrosis, Masson’s trichrome staining (Figure 5d–f) and immunohistochemical staining of COL1A1 (Figure 5g-i) were performed, which also showed no significant difference in kidneys of saline- and rMSA-treated mice. In the liver, the levels of α-SMA (Figure 5j–l), desmin (Figure 5m–o) and collagen volume fraction (Figure 5p–r) were measured to assess fibrosis levels, and no significantly differences were observed either. As for the heart, there was no significant difference in the α-SMA level by IHC assay (Figure 5s–u) and collagen volume fraction of cardiac muscle by Masson’s trichrome staining (Figure 5v–x). These phenomena suggest that exogenous rMSA treatment is safe for long-term use.

## 4. Discussion

It was well documented that the four parameters of free thiol, carbonyl, AGE, and hcy are closely related to various diseases such as diabetes mellitus, cardiovascular diseases, adiposity, and Alzheimer’s disease [10,23,29,36,37,38]. We discovered that longevity is intimately related to these four major parameters, based on which we defined the status of exogenous rMSA as “young and undamaged”. More parameters will be explored to enrich the definition of “young and undamaged” status in the future.

Results showed that young and undamaged exogenous rMSA significantly improved the grip strength and memory of mice with extended medium survival. Our separate ongoing studies show that various physiological properties can be improved, such as immune responses, metabolic processes and cardiovascular functions. Further explorations will contribute to better understanding of the mechanism of young and undamaged rMSA on longevity. It will be remarkable to see that a single young and undamaged protein (either recombinant or non-recombinant) HSA can increase the longevity of human beings, which will be initiated in the near future.

We also detected the grip strength in unmanipulated female C57BL/6N mice at 12- and 20-month-old, respectively. The saline-treated female mice at 20-month-old showed almost the same grip strength with that of the unmanipulated 20-month-old female mice, while the grip strength of rMSA-treated female mice at 20-month-old was similar to that of the unmanipulated female mice at 12-month-old (Figure S2d). These phenomena suggest that saline treatment has negligible influence on grip strength, while rMSA treatment can improve the grip strength to a younger state.

Certainly, we realized that effects of exogenous rMSA and endogenous albumin on the longevity of mice should be compared in parallel. In order to perform this experiment, endogenous albumin should be prepared from mice at different ages ranging from very young to very old, whenever exogenous rMSA was used. However, endogenous mouse serum albumin of sufficient purity is not commercially available. Moreover, at least 20,000 mice at different ages were needed to purify a sufficient amount of albumin at a purity greater than 99%, which is unethical.

Recently, Conboy group reported rejuvenation of muscle, liver, and hippocampus of mice by exchanging old blood plasma with saline containing 5% endogenous albumin [39]. Conboy group proposed that the dilution of old blood was sufficient for these rejuvenative effects, while the effects of purified commercial (fraction V) albumin was limited. Unfortunately, the intactness and damages of albumin were not reported in their study, which makes it impossible to assess the quality of their endogenous albumin.

A clinical trial whose purpose was to evaluate the beneficial effects of infusions of plasma from young donors (16–25 years old) to older adults (≥35 years old) was initiated in 2016 in the USA, but no result has been released so far (ClinicalTrials.gov Identifier: NCT02803554). Pishel group reported that the injection of the plasma from young mice (2 to 4 months) cannot improve the median lifespan of middle-aged mice (10 to 12 months) [40]. Another clinical trial initiated by Grifols (ClinicalTrials.gov Identifier: NCT01561053, NCT00742417) showed that the plasma exchange (PE) with the replacement of human serum albumin significantly slow the functional decline in patients with Alzheimer’s disease based on the Alzheimer’s Disease Cooperative Study-Activities of Daily Living (ADCS-ADL) scales. However, no significant cognitive improvement was observed in PE-treated patients measured by the Alzheimer’s Disease Assessment Scale-Cognitive (ADAS-Cog) scales [41]. Moreover, the albumin used in this study was purified from human plasma, and the intactness of albumin was not reported. We believe that young and undamaged albumin would significantly improve the cognitive ability of patients with Alzheimer’s disease.

In 2014, Wyss-Coray group reported that plasma from young mice can improve the learning and memory of old mice. Since albumin occupies about 50% of total plasma proteins, it most likely plays the most important role in this process, which was exactly what we found here. In order to achieve the maximal effect of rMSA on longevity, a variety of measures including optimal dosage, frequency, and drug delivery methods are being investigated. We predict that the concept of young and undamaged albumin increasing the longevity can also be applied to any other proteins such as immunoglobulins, fibrinogen, transferrin, transthyretin, and haptoglobin which are major plasma proteins. If so, the combination of young and undamaged major plasma proteins can further increase the longevity. Ideally, all of the young and undamaged plasma proteins altogether can increase the longevity to the largest extent.

## Data Availability

Protein accession IDs (UniProtKB): HSA: P02768, MSA: P07724, TAU: P10637, MYH7: Q91Z83, α-SMA: P62737, COL1A1: P11087, Desmin: P31001.

## Supplementary Materials

The following are available online at https://www.mdpi.com/article/10.3390/biom11081191/s1, Figure S1: Effects of rMSA injection on the levels of albumin and major blood biochemical parameters in mice; Figure S2: Effects of rMSA injection on the weight and histopathological morphology of kidney, liver and heart.

## Abbreviations

HSA	human serum albumin
AGE	advanced glycation end-product
hcy	homocysteine
rMSA	recombinant mouse serum albumin
p-tau	phosphorylated microtubule-associated protein tau
MYH7	myosin heavy chain 7
α-SMA	α-smooth muscle actin
COL1A1	collagen I
HCP	host cell proteins
qRT-PCR	quantitative RT-PCR
ELISA	enzyme-linked immunosorbent assay
GAPDH	glyceraldehyde 3-phosphate dehydrogenase
DAPI	4′,6-diamidino-2-phenylindole
DTNB	5, 5′-Dithiobis-(2-nitrobenzoic acid)
Q-TOF	Quadrupole-Time of Flight
K-S test	Kolmogorov–Smirnov test
