# Supplementary material for: Young and Undamaged rMSA Improves the Healthspan and Lifespan of Mice
Source: Biomolecules. 2021 Aug 12;11(8):1191. doi: 10.3390/biom11081191 (PMC8394218; doi:10.3390/biom11081191)
Supplement: Supplementary file 1 [file biomolecules-11-01191-s001.zip › biomolecules-1286641-supplementary.pdf]

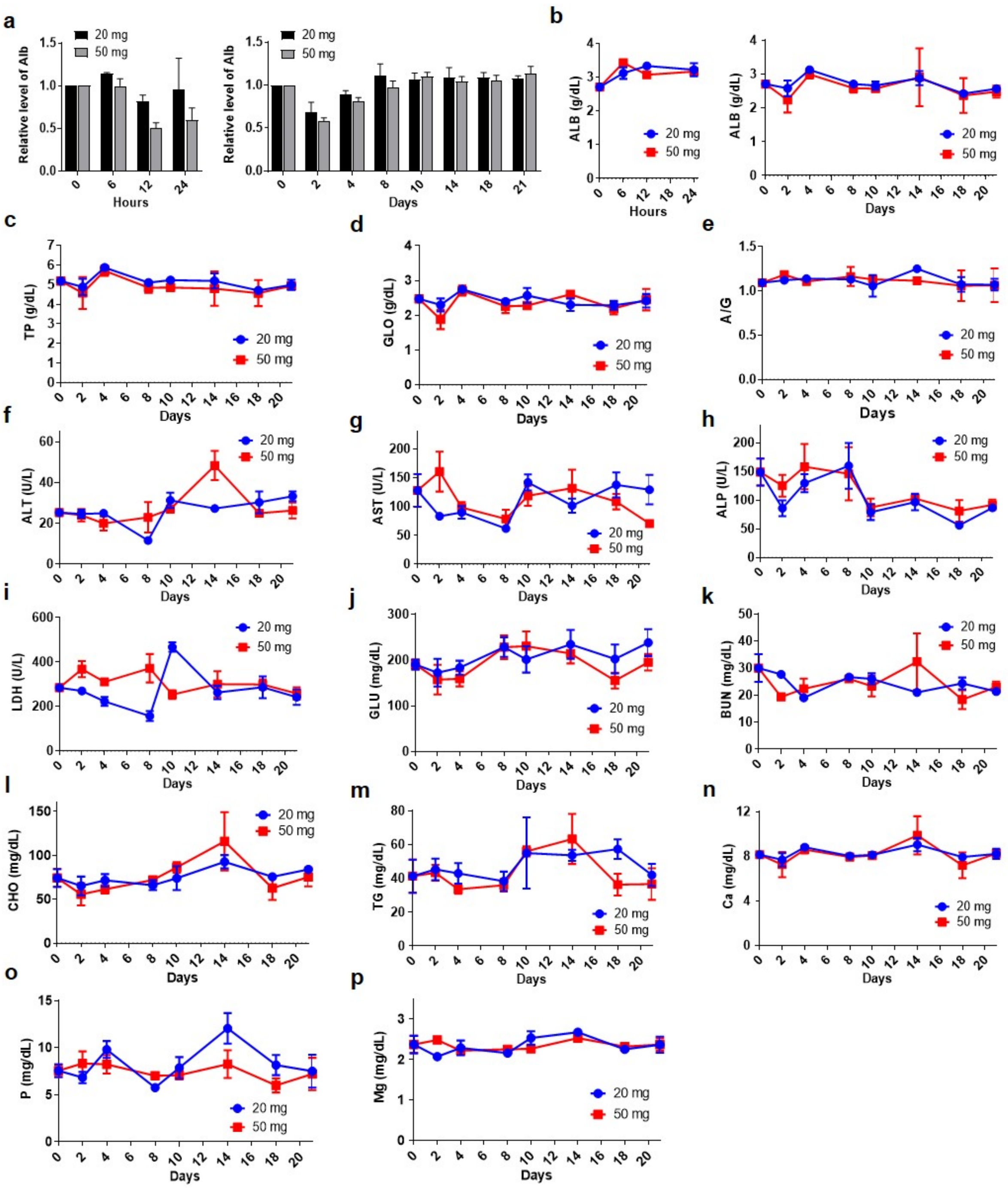

Figure S1. Effects of rMSA injection on the levels of albumin and major blood biochemical parameters in mice. (**a**) Dynamic expression levels of the albumin gene in the liver determined by qRT-PCR within 1 day (left) and 2-21 days (right) after the injection with 20- or 50 mg rMSA per mouse (n = 3). (**b**) Dynamic protein levels of the serum albumin within 1 day (left) and 2-21 days (right) after the injection with 20- or 50 mg rMSA per mouse (n = 3). (**c-e**), Dynamic total protein levels (c), total globulin levels (d), and the albumin/globulin ratio (e) within 2-21 days (right) after the injection with 20- or 50 mg rMSA per mouse (n = 3). (**f-p**) Dynamic levels of alanine transaminase (f), aspartate transaminase (g), alkaline phosphatase (h), lactate dehydrogenase (i), blood glucose (j), blood urea nitrogen (k), cholesterol (l), triglyceride (m), calcium (n), phosphorus (o), and magnesium (p) within 2-21 days after the injection with 20- or 50 mg rMSA per mouse (n = 3). All graphs represent mean with SEM.

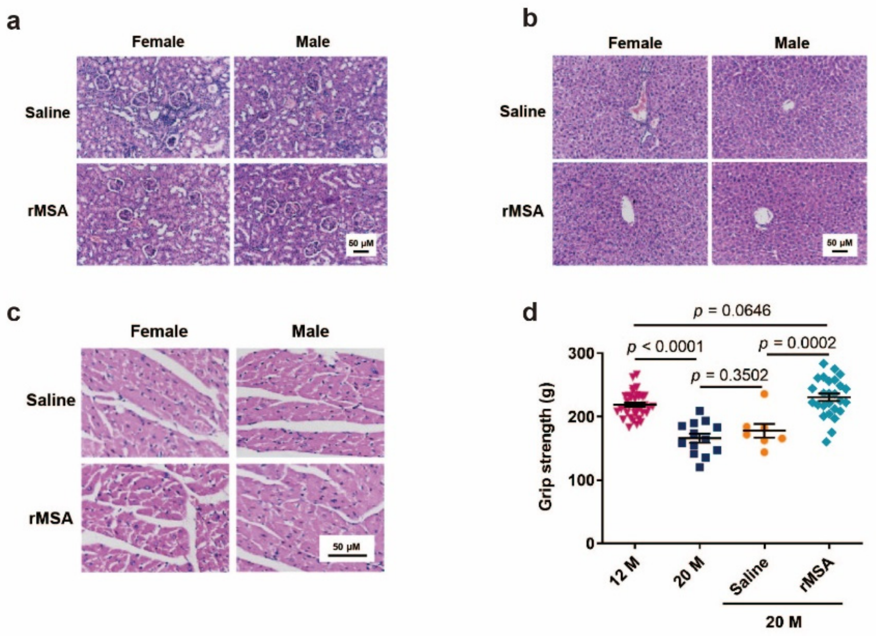

Figure S2. Effects of rMSA injection on the weight and histopathological morphology of kidney, liver and heart. (**a**) The H&E staining of the kidney in female and male mice. Scale bar, 50 μm. (**b**) The H&E staining of the liver in female and male mice. Scale bar, 50 μm. (**c**) The H&E staining of the heart in female and male mice. Scale bar, 50 μm. (**d**) The grip strength in unmanipulated and rMSA-treated female C57BL/6N mice at 12- and 20-month-old. Graph represent mean with SEM, with p values calculated by the two-tail t test.
